# How does urbanization affect the reproductive characteristics and ecological affinities of street plant communities?

**DOI:** 10.1002/ece3.5539

**Published:** 2019-08-13

**Authors:** James Desaegher, Sophie Nadot, Nathalie Machon, Bruno Colas

**Affiliations:** ^1^ Ecologie Systématique Evolution Univ. Paris‐Sud, CNRS, AgroParisTech Université Paris‐Saclay Orsay France; ^2^ Dynafor, INRA, INPT, INP‐EI Purpan Université de Toulouse Castanet Tolosan France; ^3^ Centre d'Ecologie et des Sciences de la Conservation, UMR7204 (CNRS, MNHN, UPMC) Museum National d'Histoire Naturelle Paris France

**Keywords:** citizen science, floral morphotypes, plant communities, pollination, reproductive traits, urbanization

## Abstract

Anthropogenic activities in urban ecosystems induce a myriad of environmental changes compared with adjacent rural areas. These environmental changes can be seen as series of abiotic and biotic selection filters affecting the distribution of plant species. What are the attributes of plant species that compose urban communities, compared with rural communities, as related to their ecological affinities (e.g., to temperature, humidity), and reproductive traits (e.g., entomophily, autogamy, floral morphology)? Using a floristic dataset from a citizen science project recording plant species growing spontaneously in the streets, we analyzed the distribution of species according to their ecological requirements and reproductive traits along an urbanization gradient in the Parisian region. We developed an original floral and pollinator typology composed of five floral and four pollinator morphotypes. The proportion of impervious areas, used as a proxy of urbanization, was measured at different spatial scales, to reveal at which spatial scales urbanization is selecting plant traits. We found significant differences in plant communities along the urbanization gradient. As expected with the warmer and drier conditions of urban areas, species with higher affinities to higher temperature, light and nutrient soil content, and lower atmospheric moisture were over‐represented in urban plant communities. Interestingly, all of the significant changes in plant abiotical affinities were the most pronounced at the largest scale of analysis (1,000 m buffer radius), probably because the specific urban conditions are more pronounced when they occur on a large surface. The proportion of autogamous, self‐compatible, and nonentomophilous species was significantly higher in urban plant communities, strongly suggesting a lower abundance or efficiency of the pollinating fauna in urban environments. Last, among insect‐pollinated species, those with relatively long and narrow tubular corollas were disadvantaged in urban areas, possibly resulting from a reduction in pollinator abundance particularly affecting specialized plant–pollinator interactions.

## 1 INTRODUCTION

Cities are environments in which anthropogenic activities induce a myriad of deviations from natural ecosystems (Donihue & Lambert, 2014). Both abiotic and biotic changes take place in urban areas compared with adjacent rural areas, and these changes are potentially exerting strong selective pressures on living organisms in general, and particularly for nonmobile organisms such as plants (Johnson, Thompson, & Saini, 2015). Urbanization can be seen as a series of selection filters affecting the distribution of species along rural–urban gradients. Williams et al. (2009) identified four major filters induced by urbanization, through which urban plant communities have to pass (a) habitat transformation (changes in landscape composition, such as the replacement of agricultural lands by impervious surfaces), (b) habitat fragmentation (changes in landscape structure), (c) abiotic urban environmental conditions (higher temperatures due to the urban heat island, increased water stress, and pollutions), and (d) human preference (such as the introduction of exotic plant species for ornamental purposes). The occurrence or absence of interacting organisms (in terms of herbivory, competition, or mutualism) is certainly an extra selection filter adding to these four filters. Indeed, major processes for plant population viability, such as seed dispersal by animals and pollination by insects, are entirely dependent upon the presence of the relevant animals in urban districts (e.g., Knapp et al., 2008). Through each filter, urbanization causes over‐ or under‐representation of plant species, depending on their traits. This results in differences in species occurrence and abundance between urban and rural plant communities. The impact of urbanization through selection filters also depends on the initial regional pool of plant species and the history of land use prior to the urbanization (Williams et al., 2009).

In an inductive approach, the altered distributions of biological traits in plant communities along urbanization gradients can be seen as selection signatures (Williams et al., 2009). The study of specific traits can help identify the principal selection processes occurring along these gradients and the underlying ecological mechanisms (Vallet, Daniel, Beaujouan, Rozé, & Pavoine, 2010). For example, Bechtel and Schmidt (2011) in a study of the urban heat island, and Hedwall and Brunet (2016) in a study of Swedish forests, used the spatial distributions of plant traits as bioindicators of ecological changes. In a similar way, the observation of specific plant functional traits can help predict the occurrence of mutualistic organisms. The most famous example of such prediction was made by Darwin, who suggested the existence of a then‐unknown Madagascan hawkmoth with a very long tongue, based on the observation in 1862 of an orchid whose nectar was hidden 290 mm deep in the blossom (Darwin, 1877; Kritsky, 1991).

Although biotic factors, such as pollinator availability, are recognized as important selective filters on plant species distributions (Pellissier, Pottier, Vittoz, Dubuis, & Guisan, 2010), much more attention has hitherto been given to abiotic factors such as temperature or soil composition. For example, the thriving of thermophilous plant species or plant species with affinities to dry environments in urban areas is acknowledged (Williams, Hahs, & Vesk, 2015). Soil nitrate concentration is usually higher in urban areas, a phenomenon generally attributed to atmospheric pollutant depositions that select for nitrophilous plant species (Pellissier, Rozé, Aguejdad, Quénol, & Clergeau, 2008).

Urban areas and their surrounding rural environments are good systems to study the effects of pollinator communities on plant species distribution. Indeed, several studies have shown that urbanization affects pollinator communities along urbanization gradients (Baldock et al., 2015; Deguines, Julliard, Flores, & Fontaine, 2012; Fortel et al., 2014; Geslin et al., 2016). In the region Île‐de‐France (including Paris, suburbs, and countryside), Desaegher, Nadot, Fontaine, and Colas (2018) showed that insect families of floral visitors were variously affected by urbanization and mostly in a negative way. Due to the lower abundance of most floral visitors, we can expect a smaller proportion of insect‐pollinated plant species in this urban context, as a consequence of pollen limitation, i.e., a reduced seed production caused by limited pollen availability (Knapp et al., 2008). Pollen limitation is also expected to increase the proportion of self‐compatible species with autonomous selfing capacity (Eckert et al., 2010). On the contrary, strictly self‐incompatible species should experience a reduced seed production and might be under‐represented in urban areas. To our knowledge, these hypotheses have never been tested along rural–urban gradients.

In addition to the effect on global pollinator abundances, there is a growing evidence that urbanization generates functional shifts in pollinator communities. Desaegher et al. (2018) showed that floral visitors of insect families that had a preference for nontubular corollas (generally insects with small mouthparts and considered as specialists) were rare in urbanized areas of the Île‐de‐France region (see also Geslin, Gauzens, Thébault, & Dajoz, 2013). This is consistent with the results of Deguines, Julliard, Flores, and Fontaine (2016), who showed that urbanization was associated with a shift in community composition of flower visitors toward generalist insects in France. In different contexts, two recent community‐level studies (Bergamo, Wolowski, Maruyama, Vizentin‐Bugoni, & Sazima, 2018; Fantinato, Del Vecchio, Giovanetti, Acosta, & Buffa, 2018) suggested complex interplay of facilitation and competition processes among flowering plants through pollination by insects. Both studies showed that, in a given area, plant species sharing the same pollinator guild tended to flower together (phenological synchronization), which can be interpreted as a pollinator‐mediated facilitation by increasing pollinator attraction in dense flowering patches (Sargent & Ackerly, 2008). These studies also showed that coflowering, pollinator‐sharing species tended to differ in another position in the corollas. This differentiation may allow different pollen placement on pollinator bodies, which can reduce competition among plant species and increase effective pollination (Bergamo et al., 2018; Fantinato et al., 2018). The different mechanisms by which competition for pollinators can reduce plant fitness include low frequency of pollinator visits and heterospecific pollen deposition on stigmas, and were described in detail by Waser (1978).

In the present study, we examined the distributions of plant species according to their ecological requirements and reproductive traits along an urbanization gradient. We analyzed a dataset from a French national citizen science project called “Sauvages de ma rue” (literally “wild plants of my street”) which aims at recording plant species growing spontaneously in the streets. We focused on the Île‐de‐France region (around Paris) to benefit both from a high sampling density in a full urbanization gradient and from previously published plant–pollinator studies in the region. We used the proportion of impervious areas around sampling sites as a measure of urbanization because it is obviously a major factor differentiating cities from other land uses, and because many studies have already used this measure (e.g., Ahrné, Bengtsson, & Elmqvist, 2009; Geslin et al., 2016; Pellissier, Muratet, Verfaillie, & Machon, 2012). The effect of urbanization on the occurrence of plant species according to their affinity to abiotic factors (measured by the Ellenberg indicator values) has been tested in various studies (reviewed in Williams et al., 2015). Here, for the first time we tested the effect of urbanization at different spatial scales on the affinity of plant communities to abiotic factors. These analyses are expected to reveal how the association between plant community affinities (e.g., thermophilous plants) and microclimatic conditions (e.g., urban heat islands) is sensitive to the spatial scale at which urbanization is measured.

As for abiotic factors, we studied the occurrence of plant species according to their reproductive traits at different spatial scales. Based on previous works in the Île‐de‐France region (see above), we produced two alternative hypotheses to explain the distribution of plants species according to floral morphology. The first hypothesis (H1) states that because pollinator taxa with a preference for nontubular flowers are rare in urban areas, the proportion of plant species with nontubular flowers should decrease in urban areas compared with rural areas. The alternative hypothesis (H2) states that because global pollinator abundance is low in urban areas, and because open flowers can be pollinated by a greater number of pollinator species (see Olesen, Dupont, Ehlers, & Hansen, 2007; Pellissier et al., 2010), the proportion of plant species with nontubular flowers (i.e., more generalist) should increase in urban areas. To test these two hypotheses, we developed a combined classification of flower and pollinator morphologies. Functional flower classifications in which the different flower classes are pollinated by different pollinators have already been produced in the past (Faegri & van der Pijl, 1979; Leppik, 1957; Ramirez, 2003), leading to the so‐called pollination syndromes that were shown however to have limited predictive capacities for the major pollinators of plants (Ollerton et al., 2009). Contrary to these previous flower classifications that were based essentially on flower shape or symmetry, our system is based on flower size and accessibility to pollen and nectar, which are the main floral resources for pollinators.

The question we asked in this study is as follows: what are the attributes of plant species that compose urban communities, compared to rural communities, as related to their ecological affinities (e.g., to temperature, humidity), ecological strategies (system of Grime, 1974), flowering period, reproductive traits (e.g., entomophily, autogamy), and floral morphology (e.g., tubular or nontubular flowers)? This question was addressed by testing the effect of the proportion of impervious areas on species traits at various scales around sampling sites.

## 2 MATERIAL AND METHODS

### 2.1 The “Sauvages de ma rue” protocol

The present study was based on data from a French citizen science project called “Sauvages de ma rue” aiming at collecting floristic data in France following a standardized protocol. This scientific and pedagogic project was initiated by the Centre d'Ecologie et des Sciences de la Conservation (CESCO) of the Museum National d'Histoire Naturelle (MNHN) and was promoted by the association Tela Botanica. In this program, volunteers were invited to identify and list all the spontaneous plant species growing in a street of their choice located by the GPS coordinates of the starting point of the inventory (hereafter called a sampling site). An identification guide and a mobile application were available to help the volunteers to identify the plants (http://sauvagesdemarue.mnhn.fr/biodiversit-urbaine/cl-didentification-sauvages-de-paris). Additional online identification keys and online assistance by botanists from the Tela Botanica network were also provided. The volunteers also recorded the habitat type of each plant in the street and the date of observation. For each species, a confidence degree of identification had to be specified, and pictures could be uploaded for subsequent data validation by botanists involved in the program. The program was launched in 2008 and has been running continuously since then.

### 2.2 Geographical data

A total of 1,161 different sampling sites were available in the “Sauvages de ma rue” database for the Île‐de‐France region over the years 2008–2017. This region was chosen because (a) it was one of the most densely sampled area, (b) it included seminatural habitats distant from urban influences as well as highly urbanized landscapes, and (c) studies on the distribution of floral visitors (Desaegher et al., 2018; Geslin et al., 2016) and on plant–pollinator interactions (Desaegher, Nadot, Dajoz, & Colas, 2017; Geslin et al., 2013) in this region were already available.

We used 1/5,000 GIS maps reporting land cover over the Île‐de‐France region published in 2012 by the Institut d'Aménagement et d'Urbanisme. We used seven categories of land cover: (a) forest; (b) seminatural area; (c) agricultural land; (d) water; (e) artificial open space; (f) quarries, dumps, and worksites; and (g) impervious surfaces (individual housing, group housing, business parks, facilities, and transportation infrastructures, see detailed descriptions at http://www.iau-idf.fr/). For every 1,161 sampling sites, we calculated the proportion of impervious surfaces in different radius of 50, 100, 500, and 1,000 meters using a vector layer with Quantum GIS version 2.14.13 (QGIS Development Team, 2015). The Île‐de‐France region (total area of nearly 12,000 km^2^) represented a full urbanization gradient, with samples (inventoried streets) presenting 0.5%–100% of impervious surfaces in a buffer radius of 500 m.

**Figure 1:**
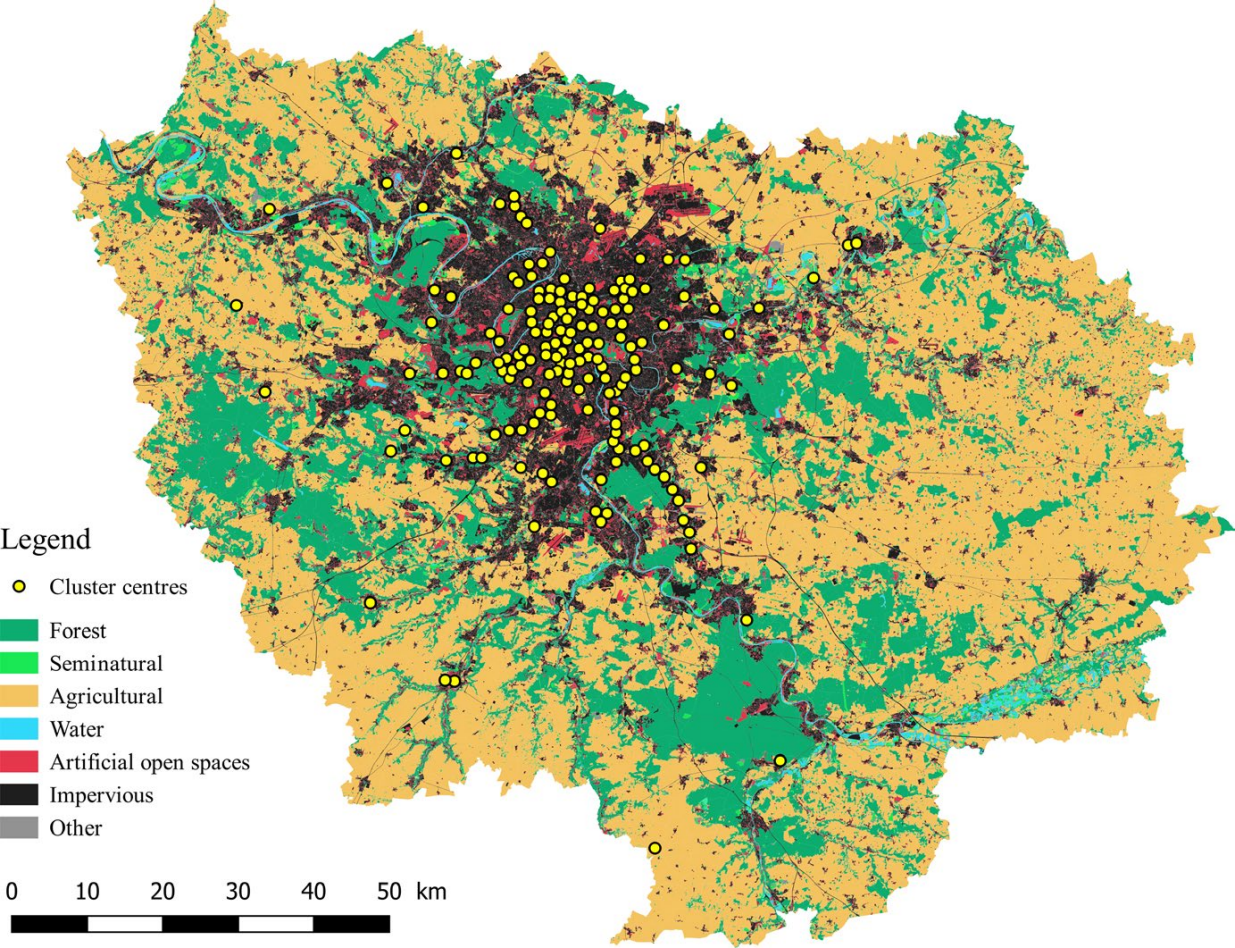
Map of the study region (Île‐de‐France) showing the distribution of the centers of the clusters of sampling sites (yellow)

Since the volunteers were free to choose their streets, the sampling sites were not geographically evenly distributed. In order to avoid spatial pseudoreplication, we grouped the sites into clusters using a Hierarchical Ascendant Classification (HAC) algorithm with R (R Core Team, 2016) as follows. All sampling sites being situated within the same region, and rather far from the poles, we first calculated the straight‐line Euclidean distance matrix between all sampling sites. Then, we performed the HAC algorithm on this matrix with the “average” method, cutting the cluster dendrogram at a height of 1,000 m. By doing so, if the average distance between the sites already included in a cluster and a new focal site was more than 1,000 m, then the focal site was not included in the cluster. We visually validated the output of this algorithm by mapping the sampling sites and the centers of gravity of every cluster using Quantum GIS version 2.14.13 (QGIS Development Team, 2015). This clustering resulted in 167 clusters of samples scattered over the study region (Figure 1). On average, the clusters were composed of 8.0 sampling sites (min. = 1, max. = 65). The mean distance among sampling sites within clusters was 206.0 m (min. = 0, max. = 752.1). The frequency distribution of several characteristics of the clusters is given in the Figure S1.

### 2.3 Biological data

Based on the identifications made by the volunteers, we established a list of plant species occurring in the study region. Species names were checked, and synonyms were replaced by the currently accepted name (http://www.theplantlist.org/). The final dataset included a total of 366 plant species.

Reproductive traits and affinity indices associated with each plant species were extracted from the online databases BiolFlor (Klotz, Kühn, & Durka, 2002) and Catminat (http://philippe.julve.pagesperso-orange.fr/catminat.htm), using the package TR8 (Bocci, 2017) with R version 3.3.2 (R Core Team, 2016). The traits extracted from BiolFlor were as follows: ecological strategy following the system of Grime (Grime, 1974), level of vegetative reproduction, level of self‐compatibility, and level of autogamy. The traits extracted from Catminat were as follows: level of entomophily, dispersal vector, beginning and end of flowering (in months) in France, affinity indices for soil granulometry and for soil organic matter, and Ellenberg indicator values (Ellenberg et al., 1992) for light, temperature, continentality, atmospheric moisture, soil moisture, soil reaction, soil nutrient content, and soil salt content, adapted to France (Ellenberg indicator values were initially developed for Central Europe).

**Figure 2:**
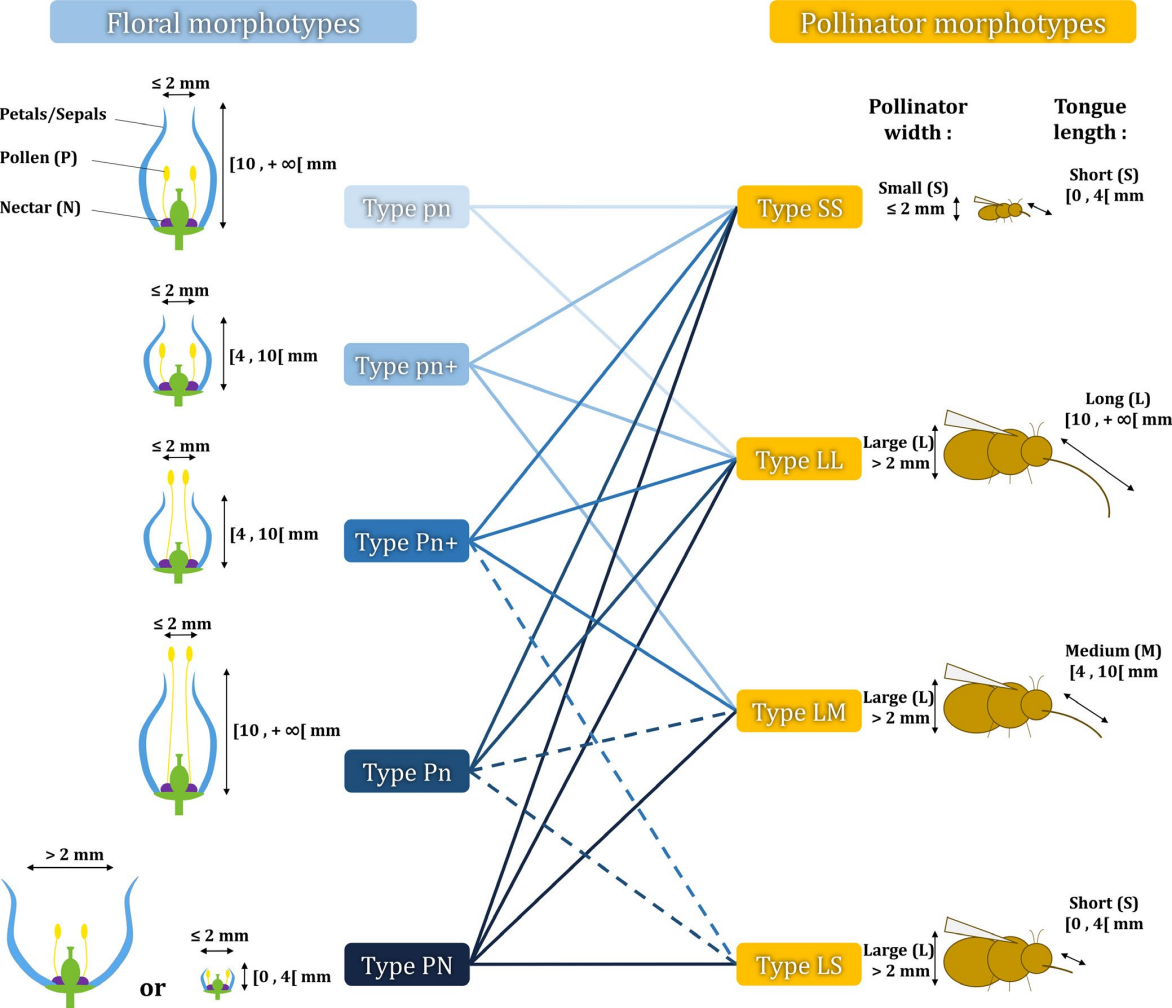
Classification of floral and pollinator morphotypes. Full lines between floral and pollinator morphotypes indicate that the pollinators can potentially access both pollen and nectar (except nectar robbing). Dashed lines indicate that the pollinators can potentially access only pollen. The drawings represent morphotypes that can belong to any of the five insect orders that include floral visitors (Hymenoptera, Diptera, Coleoptera, Lepidoptera, and Heteroptera)

We elaborated a theoretical plant and pollinator morphological typology composed of five floral morphotypes and four pollinator morphotypes (Figure 2). We considered that a pollinator can be morphologically described by the combination of (a) body width, either large (L) or small (S) (e.g., abdomen or abdomen + wings for butterflies) and (b) tongue length, either short (S), medium (M), or long (L). These arbitrary category delimitations of pollinator width and tongue length were respectively based on the range of measures recorded in entomological guides descriptions (Chinery, 2012; Leraut, Blanchot, & Hodebert, 2003) and on the floral visitor proboscis measures and natural data cuts presented in Stang, Klinkhamer, and Meijden (2006). The combination of both traits allowed us to define four pollinator morphotypes “SS,” “LL,” “LM,” and “LS” (Figure 2).

Similarly, we considered that the stamens (hence pollen) within a flower are either easily accessible to pollinators (P) or hidden within the corolla (p). Based on three categories of corolla tube length and two categories of corolla tube width, we considered that the nectar (usually stored in the bottom of the corolla) can be easily accessible (N), moderately accessible (n+), or hardly accessible (n) to pollinators. The combination of pollen and nectar accessibility allowed us to define five floral morphotypes “pn,” “pn+,” “Pn+,” “Pn,” and “PN” (Figure 2). Category delimitations of corolla length and width were chosen according to the body width and tongue length of the pollinator morphotypes. Using the morphological plant typology, each entomophilous (at least partially) plant species was assigned to one morphotype, based on botanical descriptions from floras. The theoretical relationships drawn among the plant and pollinator morphotypes (Figure 2) were based on physical constraints and on qualitative personal observations.

In addition, the presence or absence of a nectar spur and the presence or absence of tubular flowers (two floral features generally associated with nectar production) were scored for every plant species of the dataset. A plant species was considered to have tubular flowers when the corollas presented a tube longer than 4 mm, either with fused petals (e.g., the bilabiate corolla of Lamiaceae) or with free but contiguous petals forming a tube from the pollinator point of view (e.g., the papilionoid corolla of Fabaceae).

A list of the modalities of each trait and ecological affinity index (all referred to as traits hereafter) is presented in Table 1. The trait values or modalities were fixed for every species, and possible trait variation within species along the urbanization gradient was therefore not considered. We also assumed that possible mistakes in plant identification by the volunteers were mainly between plants of the same type, and were randomly distributed along the whole gradient. The list of all plant species and associated plant traits is given in Appendix S1.

**Table 1:** Summarized description of the modalities of the 21 studied plant traits. (a) Variables treated with a multinomial logistic regressions (MLR). (b) Variables treated with cumulative link mixed models (CLMM). (c) and (d) Variables treated with generalized linear mixed models (GLMM) and, respectively, a binomial and a Poisson distribution

	Trait	Modalities
a)	Floral morphotype	Type pn, pn+, Pn+, Pn, or PN (Figure 2)
	Grime's CSR ecological strategy	1) competitors (C), 2) competitors/ruderals (CR), 3) competitors/stress tolerators (CS), 4) competitors/stress tolerators/ruderals (CSR), 5) ruderals (R), 6) stress tolerators (S), 7) stress tolerators/ruderals (SR)
	Dispersal vector	1) anemochory, 2) hydrochory, 3) autochory or barochory, 4) dyszoochory or endozoochory or epizoochory or myrmecochory
b)	Level of vegetative reproduction	1) by seeds/by spores (S), 2) mostly by seeds and rarely vegetatively (SSV), 3) by seeds and vegetatively (SV), 4) mostly vegetatively and rarely by seeds (VVS), 5) vegetatively (V)
	Level of self‐compatibility	1) selfing and seed set are impossible (SI) or mostly prohibited (I+), 2) intermediate situations: (C+ and SI) or (SC and I+) or (SC, I+ and SI) or (SC and SI), 3) selfing leads to seed set (SC) or mostly leads to seed set (C+)
	Level of autogamy	1) allogamous (X) or facultative allogamous (XF), 2) intermediate situations: mixed mating (AFXF) or (AFXF and AF) or (AFXF and AO) or (AFXF and X) or (AF and XF), 3) autogamous (AO) or automixis (I) or facultative autogamous (AF)
	Level of entomophily	1) pollen not transported by insects: selfing or water or wind or (wind and selfing) or apogamy, 2) pollen partially transported by insects: (insects and selfing) or (insects and wind), 3) pollen obligatory transported by insects
	Ellenberg indicator values for: light, temperature, continentality, atmospheric moisture, soil moisture, soil reaction, nutrients and salt	From 0 or 1 to 9 or12 (Ellenberg et al., 1992)
	Affinity index for soil granulometry	1) clay, 2) intermediate, 3) silt, 4) fine sand, 5) coarse sand, 6) gravel, 7) pebbles, 8) blocks, walls slots, 9) rock plate
	Affinity index for soil organic matter	1) lithosol, arenosol, 2) carbonate mull, 3) active mull, 4) acid mull, 5) moder, 6) mor, hydromor, xeromor, 7) ranker, tangel, 8) anmoor, gyttja, 9) peat
c)	Presence or absence of tubular corolla	0) Absence or 1) Presence
	Presence or absence of nectar spur	0) Absence or 1) Presence
d)	Beginning of flowering	From January (1) to December (12)
	End of flowering	From January (1) to December (12)

### 2.4 Statistical analyses

To identify traits over‐ or under‐represented in highly urbanized areas and potentially involved in plant species adaptation, mixed models were used on the 21 traits listed in Table 1. To avoid pseudoreplication with multiple repetitions of the same plant species in a given cluster of samples, species were recorded only once in each cluster. For each species in a cluster, we calculated the mean percentage of impervious areas in a radius of 50 m around all the sampling sites where the species was found, and repeated this calculation for radii of 100, 500, and 1,000 m (hereafter called the scales of analysis). Then, we iteratively tested, for every scale of analysis, the effect of the mean percentage of impervious areas on the occurrence of every plant trait. We iteratively specified each trait variable as the response of the model, the mean percentage of impervious areas as a fixed factor, and the identity of the cluster of samples in which the traits were found as a random factor.

Three categories of statistical tests were applied depending on the trait variable modalities. Multinomial logistic regressions (MLR) were used with the package nnet (Ripley & Venables, 2016) for floral morphotype, Grime's CSR ecological strategy, and dispersal vector, three multinomial variables (Table 1a). Ellenberg indicator values, soil affinity indices, the level of vegetative reproduction, self‐compatibility, autogamy, and entomophily were considered as ordinal variables (Table 1b) and were consequently treated with cumulative link mixed models (CLMM) with logit link and flexible thresholds using the package Ordinal (Christensen, 2015a, 2015b). For both MLR and CLMM, we used a likelihood‐ratio test and pairwise comparisons were done using Wald z tests. Both presence/absence variables (“tubular corolla” and “nectar spur,” Table 1c) were analyzed with generalized linear mixed models (GLMM) using the binomial distribution (Bolker et al., 2009). The variables “beginning of flowering” and “end of flowering” (Table 1d) were analyzed with generalized linear mixed models (GLMM) using the Poisson distribution, chosen after analyses of normality and homoscedasticity on residual plots (Zuur, Ieno, & Elphick, 2010). In the case of GLMM, the significance of the mean percentage of impervious areas was tested using a Wald chi‐squared test. For the three variables related to floral morphology (“floral morphotype,” “presence or absence of tubular corolla,” and “presence or absence of nectar spur”), we restricted our analysis to the plant species recorded as at least partly entomophilous.

As recommended by Kühn and Dormann (2012), we tested for spatial autocorrelation in the residuals obtained from each of the GLMM and MLR models using the Moran test, adapted to analyse quantitative variables (Sokal & Oden, 1978). The CLMM models were excluded from these analyses because it is not possible yet to extract residuals, and raw residuals are hardly analyzable. Since the aim of this analysis was to test autocorrelation among clusters, we applied Moran tests with the mean residual value per cluster and Euclidean distance matrix among the gravity centers of the clusters. Concerning multinomial variables, the residuals obtained from the MLR model are given for each variable modalities. Since different tests were performed for a single variable (the number of tests equals the number of modalities), we adjusted *p*‐values with the Bonferroni method according to number of modalities of each variable. When autocorrelation was detected, we removed the clusters responsible for the global autocorrelation, previously identified by calculating the local indicators of spatial associations (LISA) defined by Anselin (1995). The *p*‐values associated to clusters were adjusted with the Bonferroni correction according to the number of clusters (Anselin, 1995). Models concerned with spatial autocorrelation were retested to check for the significance of factors and for the absence of autocorrelation in the new residuals.

To compare the models on any trait among the four different scales of analysis, we used the Akaike Information Criterion (AIC), allowing to identify the scale of analysis best predicting a plant trait according to the percentage of impervious area (Jackson & Fahrig, 2015). To verify that the occurrence of the five flower morphotypes along the urbanization gradient was not driven by a few taxa, we calculated the richness in families and genera for each 10% impervious areas categories in a radius of 500 m.

To inform the ecological characteristics of the Grime's CSR ecological strategies (e.g., ruderal or competitive species), we assessed the significance of the relationship between the Grime's CSR ecological strategy and all qualitative reproductive traits (level of vegetative reproduction, level of self‐compatibility, level of autogamy, level of entomophily, flower morphotypes) by performing a chi‐squared independence test on the contingency table recording the total number of plant species for each combination of the modalities of both variables.

We also tested the effect of the Grime's CSR ecological strategy, the level of autogamy, and all the other qualitative reproductive traits on the variable “end of flowering.” For this purpose, in addition to the proportion of impervious areas in a buffer of 1,000 m, either the Grime's ecological strategy or each of the qualitative reproductive traits was included as fixed factors in different GLMMs with the “end of flowering” as the response. For clarity and synthesis purpose, hereafter we focused our attention on variables that produced significant results.

## 3 RESULTS

### 3.1 Assembly of the floral dataset

The floral dataset used in this study included a total of 366 species belonging to 234 genera and 76 families (i.e., 18% of all flowering plant families). Observations were distributed in 167 spatial clusters of sampling sites, with 16 clusters presenting a mean proportion of impervious areas in a radius of 500 m comprised between “[0, 0.5],” 44 clusters between “[0.5, 0.75],” and 107 clusters between “[0.75, 1].” The high number of clusters presenting a high percentage of impervious areas is due to the fact that the program “Sauvages de ma rue” is targeted on plant species growing in streets. On average, 25.6 species were observed per cluster (see details in Figure S1).

### 3.2 Plant traits

Statistical analyses on trait frequency revealed that the significance of the effect of the percentage of impervious areas was dependent upon the scale of analysis (Table 2). However, there were no opposite significant effects among the different scales for a single trait. Among the 21 traits examined, eleven traits were significantly affected by the percentage of impervious areas at least at one scale of analysis. Only two traits, namely “presence or absence of tubular corolla” and “end of flowering,” displayed autocorrelation of residuals with for instance, respectively, seven and eight clusters responsible for the global autocorrelation, with impervious areas calculated in a buffer of 500 m. At all scales, *p*‐values were corrected (Table 2) and no autocorrelation was found after removing these autocorrelated clusters.

**Table 2:** Results of the statistical models testing for the effect of the mean percentage of impervious areas around the sampling sites in a buffer of 50, 100, 500, and 1,000 m on the 21 plant traits

Model	Plant trait	50 m	100 m	500 m	1,000 m
MLR	Floral morphotypes	<.001***	<.001***	.09	.055
	Grime's CSR ecological strategy	<.001***	<.001***	.004**	.002**
	Dispersal vector	.981	.929	.177	.03*
CLMM	Level of vegetative reproduction	.003 (−)**	.008 (−)**	.004 (−)**	**<.001** (−)***
	Level of self‐compatibility	.012 (+)*	.003 (+)**	.005 (+)**	.02 (+)*
	Level of autogamy	.002 (+)**	<.001 (+)***	<.001 (+)***	<.001 (+)***
	Level of entomophily	.713 (−)	.209 (−)	**.06 (−)**	NA
	Ellenberg for light	.458 (−)	.664 (−)	.33 (+)	.002 (+)**
	Ellenberg for temperature	.08 (+)	.093 (+)	.008 (+) **	<.001 (+)***
	Ellenberg for continentality	NA	NA	NA	.204 (−)
	Ellenberg for atmospheric moisture	.875 (+)	.607 (−)	.037 (−)*	<.001 (−)***
	Ellenberg for soil moisture	NA	**.325 (−)**	**.092 (−)**	NA
	Ellenberg for soil reaction	NA	.078 (+)	NA	NA
	Ellenberg for nutrient content	.244 (+)	.115 (+)	.117 (+)	.015 (+)*
	Ellenberg for salt	.220 (+)	NA	NA	NA
	Affinity index for soil granulometry	NA	NA	.212 (+)	NA
	Affinity index for soil organic matter	NA	**.288 (−)**	NA	**.499 (−)**
GLMM Binomial	Presence or absence of tubular corollas	.514 (−)	.861 (+)	.61 (+)	**.503 (+)**
	Presence or absence of nectar spur	**.084 (+)**	**.11 (+)**	**.15 (+)**	.497 (+)
GLMM Poisson	Begin of flowering	**.267 (−)**	**.523 (+)**	**.868 (+)**	**.478 (+)**
	End of flowering	.330 (+)	.2383 (+)	**.0835 (+)**	.0418 (+)*

Notes

Figures in bold indicate the best model according to Akaike Information Criterion (AIC) i.e., with a ΔAIC > 2 with the other models (Jackson & Fahrig, 2015). Asterisks indicate the level of statistical significance (*p*‐value: 0 ≤ *** < .001 ≤ ** < .01 ≤ * < .05). Signs of the estimate are between brackets. NA means that the CLMM model returned errors.

**Figure 3:**
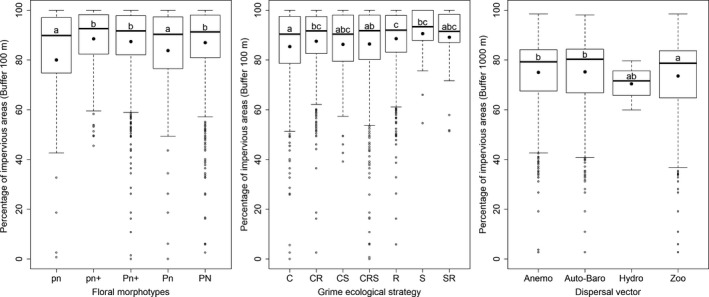
Boxplots representing the relationships between each of the three multinomial variables and the mean percentage of impervious areas around the sampling sites at the best scale of analysis according to AIC. Left: floral morphotypes with the codes defined Figure 2. Middle: Grime ecological strategies with “C” for competitors, “R” for ruderals, and “S” for stress tolerators. Right: Dispersal vectors with “Anemo” for anemochory, “Hydro” for hydrochory, “Auto‐Baro” for autochory or barochory and “Zoo” for zoochory. Black dots represent the means for each modality. For each variable, modalities sharing the same letters do not significantly differ

Based on multiple comparisons of the occurrences of flower morphotypes, we showed that at small scales (50 and 100 m), the increasing percentage of impervious areas was significantly associated with a reduced frequency of morphotypes pn and Pn, with nectar hardly accessible to pollinators (Figure 2, Table 2 and Figure 3). A higher proportion of impervious area was significantly associated with reduced occurrence of zoochorous compared with autochorous and anemochorous species, but only at the largest scale of analysis (Table 2 and Figure 3). Last, a higher proportion of impervious area at all scales was significantly associated with a reduced occurrence of competitive plant species (C‐strategists) and an increased occurrence of ruderal species (R‐strategists) (Table 2 and Figure 3).

The increasing percentage of impervious areas was significantly associated with a reduced level of plant vegetative reproduction and a reduced affinity for humid atmosphere, especially at large scales (Table 2). There was a marginally significant negative effect on the level of entomophily (*p*‐value = .06 with buffer 500 m). On the opposite, increasing percentage of impervious areas was significantly associated with high levels of self‐compatibility, autogamy, and affinity for high temperatures, for nutrient‐rich environments, and for unshaded environments. No significant results were obtained for the remaining plant ecological affinity nor for the occurrence of tubular corolla and nectar spur.

Plants having a flowering period ending later and a longer flowering period (no significant differences for the beginning of flowering) were favoured in sites surrounded by a higher proportion of impervious areas (at larger scales: buffer 500–1,000 m, Table 2). However, when the variable accounting for plant ecological strategies was included in the GLMM model together with the proportion of impervious areas (buffer radius 1,000 m) in order to explain the flowering end, a significant effect of the ecological strategies was detected (*p*‐value < .001) and the effect of impervious areas became nonsignificant (*p*‐value = .128). Similarly, when the variable accounting for the level of autogamy was included in the GLMM model together with the proportion of impervious areas (buffer radius 1,000 m) to explain the flowering end, a significant effect of the level of autogamy was detected (*p*‐value = .002) and the effect of impervious areas became nonsignificant (*p*‐value = .087). Pairwise comparisons in both GLMM models revealed that the R‐strategists (ruderal species) had a significantly longer and later flowering period than the S‐strategists and C‐strategists, and that the “autogamous or facultative autogamous” species had a significantly longer and later flowering period than the species with “intermediate mating systems” and “allogamous or facultative allogamous” species. Furthermore, the chi‐squared independence test used to assess the relationship between the Grime's CSR ecological strategy and the level of self‐compatibility revealed a significant correlation between both traits (*p*‐value < .001). The R‐strategists were significantly more frequently composed of “autogamous or facultative autogamous” species, C‐strategists were significantly more frequently composed of “allogamous or facultative allogamous,” and S‐strategists were significantly more frequently composed of species with “intermediate mating” systems.

## 4 DISCUSSION

In the present study, we revealed significant changes in the distribution of reproductive characteristics and ecological affinities of street plants along the urbanization gradient in the Île‐de‐France region. Thermophilous species and species tolerant to drought conditions and unshaded environments were significantly associated with high proportions of impervious areas at the largest scale of analysis. We also showed that urbanization acted as a selective filter on plant species according to their dispersal vector and flowering time, by disfavoring zoochorous species and favoring late‐flowering species. Our most striking result was that high proportions of impervious areas were associated with an increased frequency of autogamous and self‐compatible species and a decreased frequency of species with long and narrow tubular corollas, suggesting a pollination filtering, acting on plant species distribution along the urbanization gradient.

### 4.1 Abiotic ecological preferences

The relationship between urbanization and the ecological affinity indices (accounting for abiotic ecological conditions) found here is consistent with the recent synthesis of Williams et al. (2015). A higher frequency of thermophilous species in urban environments had already been observed two decades ago (Pyšek, 1998) and was further reported in later studies (Williams et al., 2015). The thriving of native and alien thermophilous plant species (e.g., archaeophytes from Mediterranean regions; Pyšek, 1998) can be explained by the urban heat island (UHI). The UHI can also induce a higher evaporation resulting in a drier atmosphere, potentially responsible for the reduced occurrence of plant species with a strong affinity to atmospheric moisture. Although depending on the context of urbanization, the higher occurrence of plant species preferring unshaded environments is consistent with previous studies (Thompson & McCarthy, 2008; Williams et al., 2015). The higher frequency of plant species with high affinity to nutrient‐rich soils in urban environments found here is also often reported (Kalusová, Čeplová, & Lososová, 2017; Thompson & McCarthy, 2008; Williams et al., 2015) and can be explained by higher nitrate and lower ammonium concentration in the soil (Pellissier et al., 2008). Interestingly, all significant Ellenberg indicators are scale sensitive (according to Jackson & Fahrig, 2015) with a maximum effect at the largest scale of analysis (buffer radius = 1,000 m). To our knowledge, this has never been reported before. This result could be related to the fact that these indicators are associated with the installation of microclimatic conditions (e.g., UHI, atmosphere drying up, atmospheric pollutions) that require a minimum spatial extent to take place and stabilize because of atmospheric convections and diffusions.

### 4.2 Ecological strategies and flowering phenology

Concerning the distribution of Grime's ecological strategies along the urbanization gradient, we found a dominance in R‐strategists in urban areas, and a dominance of C‐strategists in periurban or suburban areas, as previously observed (Pellissier et al., 2008). This is not totally surprising since Grime (1974) defined R‐strategists as tolerant to disturbances damaging the vegetation (e.g., grazing, mowing, trampling, ploughing), which obviously occur frequently in urban areas. The S‐strategists are defined as tolerant to continuous and severe stresses inhibiting plant development, such as nutrient deficiencies, shading, or desiccation (Grime, 1974). The distinction between R‐strategists and S‐strategists is sometimes difficult. Indeed, certain environmental factors, such as drought, are more pronounced in urban areas than in rural areas (Lambrecht, Mahieu, & Cheptou, 2016) and can induce both severe stresses and disturbances damaging the vegetation, accounting for the intermediate response to urbanization of the S‐strategists in our study.

Many studies have shown that plants tend to flower earlier in warmer conditions (Fitter & Fitter, 2002; Franks, Sim, & Weis, 2007; Roetzer, Wittenzeller, Haeckel, & Nekovar, 2000). Consistently, Neil, Landrum, and Wu (2010) showed that there was a higher proportion of plant species with advanced flowering in (warmer) urban areas compared with the proportion of species with delayed flowering. We thus expected to observe an increase in early‐flowering species along with an increasing percentage of impervious areas in our study, but on the opposite our results revealed an increase in late‐flowering species as urbanization increased. Several explanations can hold for this unexpected result. First, unlike what was done in other studies (Fitter & Fitter, 2002; Franks et al., 2007; Roetzer et al., 2000) we did not study the shifting of phenology within species, we studied species composition according to their reported flowering period, and it has to be noted that both late‐flowering and early‐flowering species can flower earlier when urbanization increases. Second, urbanization gradients are associated with a syndrome of environmental changes including many factors other than temperature increase (Donihue & Lambert, 2014; Johnson et al., 2015). Increased water stress or various pollutions (higher concentrations in CO2 or in volatile organic compounds, decreased UVB, higher light pollution) may also have an influence on flowering phenology (Neil & Wu, 2006), which could be different from one city to the other. Third, it is possible that long and late‐flowering species were indirectly selected in urban areas by hitch‐hiking (Smith & Haigh, 1974) along with other plant characteristics under direct selection. Indeed, our results revealed that the flowering period was also related to the ecological strategies of the plants and to the degree of autogamy, which is consistent with a significantly longer and later flowering period of R‐strategists compared with S‐ and C‐strategists recently shown by Novakovskiy, Maslova, Dalke, and Dubrovskiy (2016).

### 4.3 Reproductive traits and flower morphology

Munoz, Violle, and Cheptou (2016) observed recently from a 2,000‐species dataset that ruderal species were mostly autogamous, while competitive species were more often allogamous, and stress‐tolerant species tended to have a mixed mating system. Our results on species traits along an urbanization gradient are entirely congruent with these findings. The observed interdependence between the ecological strategies and the degree of autogamy (R‐strategists being more frequently autogamous or facultative autogamous) probably contributes to the advantage of R‐strategists in urban areas over the C and S‐strategists. Indeed, urban plant communities are likely more subject to pollen limitation than rural plant communities, which selects for autonomous selfing as a means to ensure reproduction (Eckert et al., 2010). Pollen limitation can result from various causes, such as reduced pollen availability (due, e.g., to a small number of plants in the population), inefficient pollen transport (e.g., low pollinator abundance in the area, few pollinator visits), or fewer pollen grains reaching the ovules (*e.g*., low rate of pollen germination, pollen‐tube attritions due to self‐incompatibility mechanisms, cool temperatures; Harder & Aizen, 2010). Entomophilous plants were less frequent in urban areas compared with more rural areas in our study region, as already shown in Germany (Knapp et al., 2008). This may reflect a reduced pollinator abundance resulting in insufficient pollination for many plant species growing in urbanized areas, disadvantaging entomophilous plants. This interpretation is consistent with the decrease in wild bee abundance and richness, and the decreased number of plant–pollinator interactions with increasing percentage of impervious areas observed in the Parisian region (Desaegher et al., 2018; Geslin et al., 2013, 2016). As a likely consequence of pollinator limitation, Pellissier et al. (2012) experimentally evidenced a decreased fruit production in urban study sites compared with periurban sites in *Lotus corniculatus*, in the region.

The decrease in the occurrence frequency of species bearing flowers with long and narrow tubular corollas (pn and Pn morphotypes in Figure 2, both with restricted access to nectar) with increasing urbanization corresponds to the hypothesis H2 stated in the introduction (expected increase in the proportion of generalist plant species with nontubular flowers in urban areas because of lower pollinator abundance). Flower openness, which determines the access to floral resources, is often associated with the level of generalization (i.e., possible pollination by a greater number of pollinator species), and the reduction of pollinator abundance in urban areas should favor generalist plant species and in turn result in an increase of the proportion of nontubular flowers. According to the theoretical relationships among plant and pollinator morphotypes proposed in Figure 2, morphotypes “pn” and “Pn” are the most specialized flowers for nectar accessibility because of their long tubular corrolas. It is possible that species bearing these types of flowers are more affected than the others by a general decrease in the abundance of pollinators. Alternatively, it is possible that “pn” and “Pn” floral morphotypes are affected in cities by a particular decrease in insects with morphotypes “SS” and “LL.” Both morphotypes can access hidden nectar, due to their small body (“SS”) or their long tongue (“LL”) (Figure 2), and are therefore more likely to efficiently pollinate these flowers. In particular, small‐bodied floral visitors are more likely to be affected by urbanization because of reduced nesting opportunities (they are often ground‐nesting) and limited flight capacities among suitable sites (Gathmann & Tscharntke, 2002; Geslin et al., 2016; Greenleaf, Williams, Winfree, & Kremen, 2007).

In the present study, we did not take into account possible trait variation or local adaptation through natural selection within species along the urbanization gradient, unlike in other studies (Brys & Jacquemyn, 2012; Cheptou, Carrue, Rouifed, & Cantarel, 2008; Desaegher et al., 2017; Thompson, Renaudin, & Johnson, 2016). However, for several traits the BiolFlor and Catminat databases specify multiple modalities reflecting plant trait variability (e.g., level of self‐compatibility, level of entomophily). Since we gathered different trait modalities initially recorded in the databases, the information contained in the analyzed variables was conservative regarding the variability of traits along the urbanization gradient. Another potential caveat of our analyses is that we did not formally take into account potential phylogenetic constraints in the distribution of traits along the urbanization gradient, which may bias correlations between trait distributions and the level of urbanization (Pellissier et al., 2010). Nevertheless, we verified that the significant frequency reduction of morphotypes “pn” and “Pn” with increasing urbanization was not due to a single family or genus. For example, the clusters associated with a proportion of impervious areas comprised between “[0, 0.1],” “[0.4, 0.5],” and “[0.9, 1]” in a radius of 500 m were associated with, respectively, three, four, and six families. Morphotypes “pn” and “Pn,” compared with the three other morphtotypes, could have been particularly subject to such biases because they were less frequent in the study region.

Overall, the over‐representation of autogamous and self‐compatible species suggests the existence of pollen limitation in urban areas, probably due to a decreasing abundance of pollinators while urbanization increases. The lower frequency of the most specialized floral morphotypes observed in highly urbanized areas may be due to a specific reduction in pollinators visiting this floral morphotypes or due to the fact that specialized plant species are particularly prone to be affected by a general reduction in pollinator abundance. This latter point calls for studies investigating the functional relationships between plants and pollinators along urbanization gradients. The morphotype classification elaborated in the present study can provide a functional framework for such studies. Estimating the frequencies of the interactions or even the pollen flow among the plant and pollinator morphotypes would add valuable information to this network.

The use of citizen science projects based on plant or pollinator inventories seems promising to study functional relationships, in spite of unavoidable occasional misidentifications. This drawback is compensated by the large and extremely valuable quantity of sampling sites, and the study of functional interactions does not necessarily require accurate taxonomic resolution. We believe that the mapping of plant traits (e.g., floral morphotypes) have the potential to efficiently detect ecological trends and could help identify the most problematic environmental disturbances or urban planning for biodiversity persistence. This information could also help implement sustainable plant species introductions in urban green spaces (e.g., parks, roofs or green walls), adapted to the abiotic and biotic conditions imposed by the urban environment, and favoring wild pollinators in cities.

## CONFLICT OF INTEREST

None declared.

## AUTHOR CONTRIBUTIONS

This study constitutes a part of the doctoral thesis of JD under the supervision of BC and SN. All authors contributed to the writing of the manuscript and gave approval for publication.

## Data Availability

The raw data of species occurrence can be downloaded in the section “Les données d'observation” under the project name “Sauvages de ma rue” at: http://www.tela-botanica.org/page:telechargement_des_donnees?langue=fr
