# Supplementary material for: How does urbanization affect the reproductive characteristics and ecological affinities of street plant communities?
Source: Ecol Evol. 2019 Aug 13;9(17):9977–89. doi: 10.1002/ece3.5539 (PMC6745663; doi:10.1002/ece3.5539)
Supplement: Supplementary file 1 [file ECE3-9-9977-s001.docx]

**Online supplementary material 1: Precisions about the clusters of samples**

**Journal: Ecology and evolution**

**Title: How does urbanisation affect the reproductive characteristics and ecological affinities of street plant communities?**

James Desaegher^1,2^*, Sophie Nadot^1^, Nathalie Machon^3^, Bruno Colas^1^

Affiliation of authors:

^1^ Ecologie Systématique Evolution, Univ. Paris-Sud, CNRS, AgroParisTech, Université Paris-Saclay, 91400 Orsay, France

^2^ Dynafor, Université de Toulouse, INRA, INPT, INP-EI Purpan, Castanet Tolosan, France

^3^ Centre d’Ecologie et des Sciences de la Conservation, UMR7204, (CNRS, MNHN, UPMC), Museum national d’Histoire naturelle, 55 rue Buffon, 75005 Paris, France


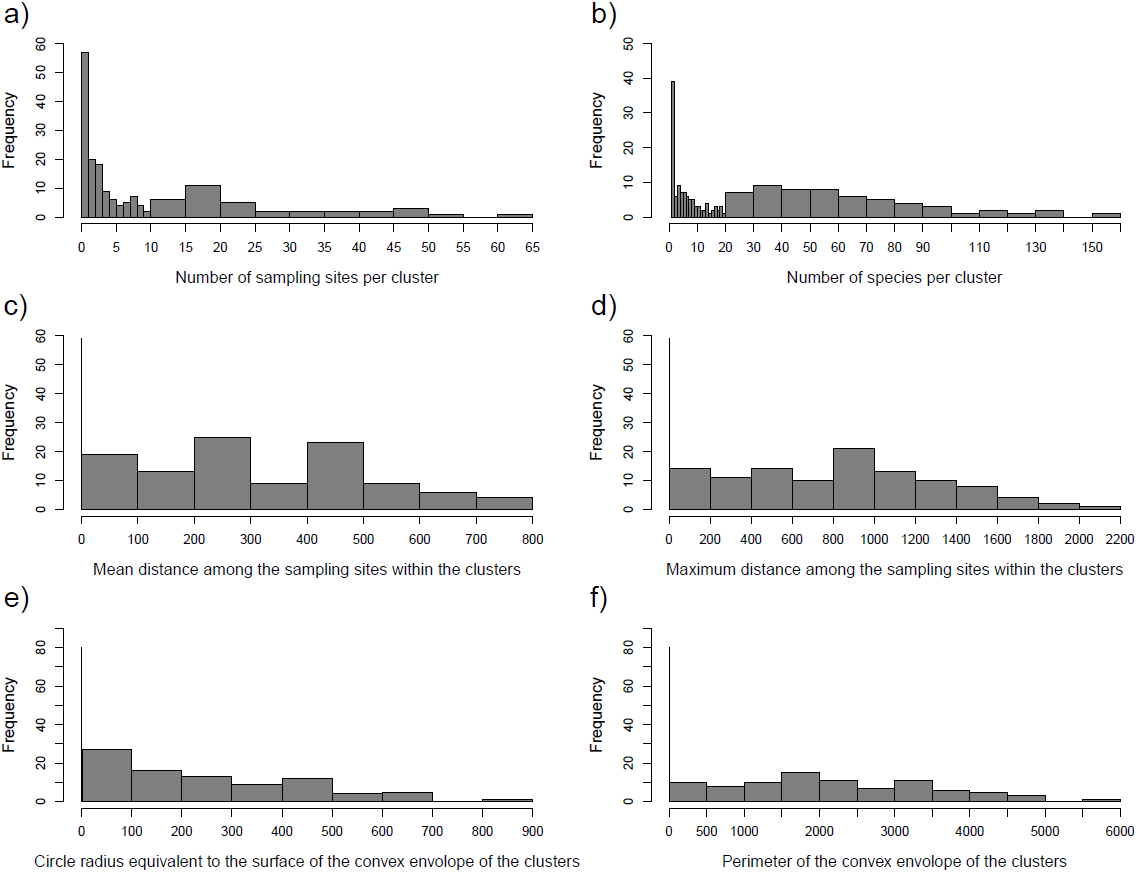


**Figure 1**: Histograms of the characteristics of the clusters of samples in the study region. For the histograms c), d), e), f) the vertical bar above zero represent the number of clusters with a value of zero.
